# Tunable Adhesion for Bio-Integrated Devices

**DOI:** 10.3390/mi9100529

**Published:** 2018-10-18

**Authors:** Zhaozheng Yu, Huanyu Cheng

**Affiliations:** 1 Department of Engineering Science and Mechanics, The Pennsylvania State University, University Park, State College, PA 16802, USA; zqy5106@psu.edu; 2 Materials Research Institute, The Pennsylvania State University, University Park, State College, PA 16802, USA

**Keywords:** bio-integrated devices, tissue adhesives, tunable adhesion, dry/wet conditions, soft biological tissue

## Abstract

With the rapid development of bio-integrated devices and tissue adhesives, tunable adhesion to soft biological tissues started gaining momentum. Strong adhesion is desirable when used to efficiently transfer vital signals or as wound dressing and tissue repair, whereas weak adhesion is needed for easy removal, and it is also the essential step for enabling repeatable use. Both the physical and chemical properties (e.g., moisture level, surface roughness, compliance, and surface chemistry) vary drastically from the skin to internal organ surfaces. Therefore, it is important to strategically design the adhesive for specific applications. Inspired largely by the remarkable adhesion properties found in several animal species, effective strategies such as structural design and novel material synthesis were explored to yield adhesives to match or even outperform their natural counterparts. In this mini-review, we provide a brief overview of the recent development of tunable adhesives, with a focus on their applications toward bio-integrated devices and tissue adhesives.

## 1. Introduction

Although adhesion has long been studied, early efforts focused on the contact between stiff materials [1]. Due to the emerging interest in reconfigurable systems [2] and bio-integrated devices [3,4], adhesion that involves a soft material with different levels of adhesion strength or even a tunable range started attracting attention. Soft materials of interest range from synthetic polymers to biological tissues [5]. Adhesion involving soft materials could be affected by the surface structure/morphology, the deformation of soft materials, and wet/dry conditions, among many others [6,7,8]. The strategies to design and achieve various levels of adhesion strength can be achieved through structural designs or material innovations. The rapid development in both these classes is greatly promoted by bio-inspiration from several marvelous animals (e.g., gecko, octopus, and mussel), which shed light on the effects of surface roughness, the directionality of the adhesive, and surface chemistry [6,9,10,11]. Although adhesion is greatly modulated by the properties of the adhesive layer, it is also affected by the target substrates due to interaction at the adhesive–substrate interface; thus, the adhesive has to be specifically designed for each application [12]. 

When it comes to adhesion to biological tissues, tunable adhesion is of great importance. For instance, strong adhesion to the wound edge is expected in a tissue adhesive to suture the wound [13]. Upon completion of wound healing, a weak adhesion is then desirable for easy removal of the adhesive. Tunable adhesion is also one essential step for realizing repeatable use, as easily removed adhesive could be sanitized and prepared for further use. Current commercial tissue adhesives such as Dermabond^®^ [14] are designed for one-time use. However, when combined with tunable properties, tissue adhesives can be used as an alternative to surgical sutures in clinical practice to eliminate the need for stitch removal. As a decrease in adhesion is observed following multiple uses of the adhesive, strategies to minimize such a decrease need to be explored [15]. 

When integrated with functional sensing components and actuators, soft adhesives could provide a natural interface to enable bio-integrated devices (e.g., flexible displays [16,17,18], wellness monitors [19,20,21,22,23], and therapeutic devices [24,25]). The soft nature of the adhesive could minimize discomfort, while strong adhesion improves signal transfer through an enhanced signal-to-noise ratio [26]. Reversible adhesion also allows multiple uses of bio-integrated electronics. Moreover, the capability to modulate interfacial adhesion finds application in the development of transfer printing [27,28,29,30], which is widely used for the assembly of heterogeneous materials [31] with applications from wearable devices [3,32,33,34] to biodegradable sensors [35,36,37,38,39]. 

In this mini-review, we firstly provide a brief overview of the structural design for adhesives with applications mostly in the dry environment. As extensive review articles exist for dry adhesives [8,40,41,42], only selected key developments are highlighted here. Next, we discuss material innovations for using adhesives in the wet environment, which are largely based on bio-inspiration from mussels. As special considerations have to be given to the application of adhesives on biological tissue surfaces, we then highlight several recently developed techniques for such applications.

## 2. Structural Design for Dry Adhesion

**Figure 1:**
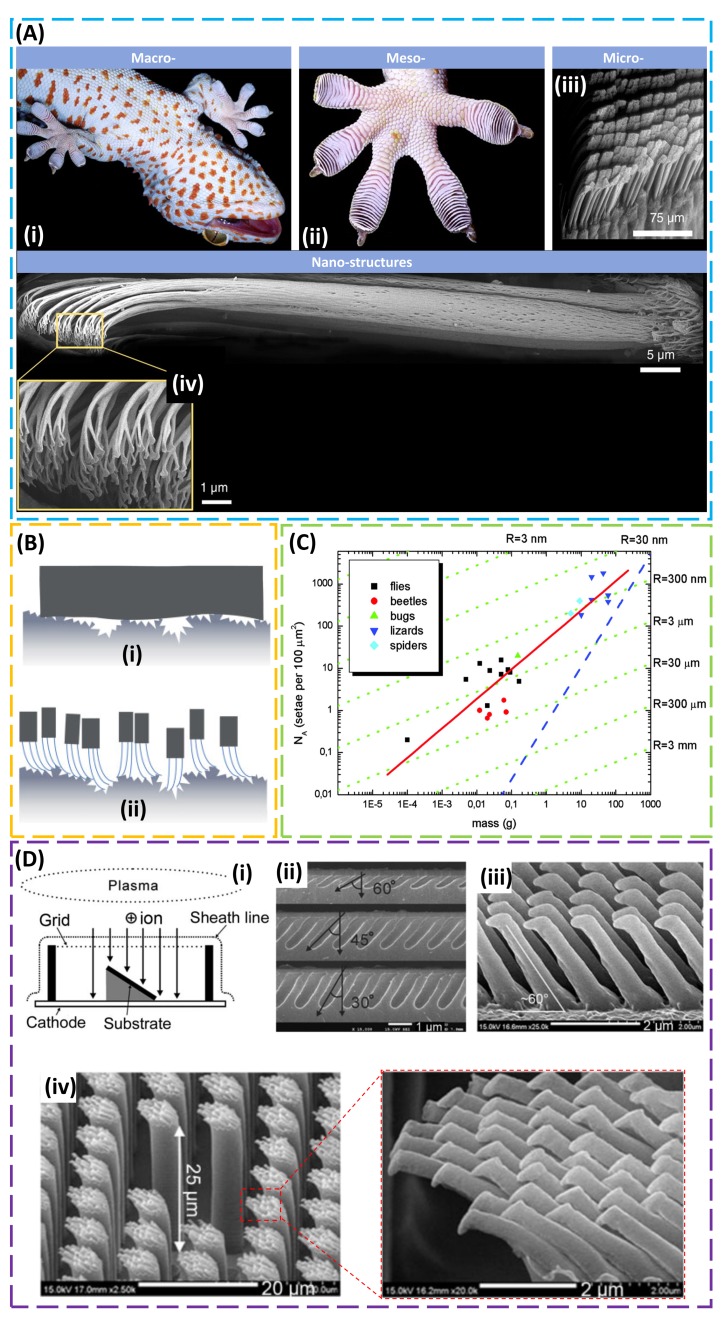
Gecko-inspired dry adhesive. (**A**) (**i**) A close-up view of a gecko climbing a glass wall and (**ii**) mesostructure of gecko toe pad with (**iii**) microscale array of high-aspect-ratio setae structure. (**iv**) Cryo-SEM view of a single seta (ca. 110 µm in length and 4.2 µm in diameter) that branches at the tips into 100–1000 more structures known as spatulas. Reproduced with permission from Reference [56]; Copyright 2006, The Company of Biologists. (**B**) Schematic illustrations of adhesives with (**i**) a relatively flat surface and (**ii**) an array of fibrils in contact with a rough surface. Reproduced with permission from Reference [49]; Copyright 2010, John Wiley and Sons. (**C**) Dependence of the terminal element density (N_A_) of the attachment pads on the body mass (m) in hairy-pad systems of diverse animal groups. The red line that fits all data corresponds to the self-similarity criterion with a slope of ~0.67. The green lines (slope of ~0.33) correspond to the model with a curvature invariance of contacts with radius *R*. The blue line shows the approximate limit of maximum contact for such attachment devices. Reproduced with permission from Reference [47]; Copyright 2003, United States National Academy of Sciences. (**D**) (**i**) A schematic showing the mechanism of a plasma sheath with a Faraday cage, where ions are incident on the substrate surface in a direction normal to the grid plane for an angled etching in the silicon substrate. (**ii**) SEM images of polySi etch profiles with angles of 30°/45°/60°, and (**iii**) polyurethane acrylate (PUA) nano-hairs formed on a poly(ethylene terephthalate) (PET) film substrate with a slanted angle of 60°. (**iv**) SEM image of two-level hierarchical PUA hairs with well-defined high aspect ratio over a large area using two-step ultraviolet (UV)-assisted capillary force lithography; the inset shows angled nano-hairs on the tip. Reproduced with permission from Reference [9]; Copyright 2009, United States National Academy of Sciences.

Due to its remarkable ability to climb rapidly up a variety of vertical surfaces (Figure 1A (i)), the gecko inspired researchers to uncover the underlying mechanisms behind its significant enhanced, highly robust and repeatable, and reversible adhesion. Observation of the pad area (Figure 1A (ii)) shows nearly 500,000 keratin setae (pillars) (Figure 1A (iii)), with each seta consisting of branches of spatulas that are approximately 200 nm in diameter and 20–60 µm in length (Figure 1A (iv)) [11]. Experimental evidence confirmed that the dry adhesion of gecko setae results from van der Waals forces rather than mechanisms associated with a high surface polarity such as capillary adhesion [43], which indicates that the exceptional adhesion is merely a result of the size and shape of the setae tips. The direct observation of the van der Waals interaction indicates that the adhesion is not affected by the surface chemistry, and repeatable use is possible [40]. In order to reveal the role of the van der Waals interaction on the enhanced adhesion observed in the gecko pad [41,44,45], an array of biomimetic microscopic fibrils on an elastic support was created [41,46]. In direct contrast to a flat surface that only has limited contact to the target substrate with a microscale surface roughness, the array of fibrils with a high aspect ratio in a dense arrangement [47] was observed to form intimate contact with the target substrate due to its low effective Young’s modulus and increased effective contact area, especially when a preload was applied (Figure 1B) [48,49]. The principle of contact mechanics was further applied to illustrate that contact splitting (i.e., reducing the radius of the fibril) yielded substantially improved adhesion, and the scaling was found to be applicable to animals differing in weight by six orders of magnitude (Figure 1C) [45,47,50]. The use of soft polymers in most biomimetic systems helps increase the adhesion, but their tacky nature also makes them more susceptible to particulate fouling; thus, a hydrophobic surface with the capacity for self-cleaning is desired. In fact, a fibrillar adhesive can partially transfer particles in a certain size range from its surface to the clean substrate and recover ca. one-third of its shear adhesion [51], as observed in gecko setae [52]. In the practical application where defects commonly exist, the fibrillar structure also localizes the contact failure at individual fibrils and minimizes the effect on contact adhesion, thereby increasing the defect tolerance. Moreover, the adhesion is also affected by the underlying supports. By peeling a polydimethylsiloxane (PDMS) substrate patterned with different hexagonal arrays of cylindrical pillars (to mimic fibrils) from an acrylic adhesive, the enhancement in the adhesion was shown to be more than the increase of the contact area, and this was attributed to the deformation of the underlying support [6]. The fibrillary structure can also be used directly beneath the existing viscoelastic adhesive film (e.g., pressure-sensitive adhesives) to change the dissipative crack trapping and the stress field in the viscoelastic layer for enhanced adhesion [53].

Due to the need for locomotion [54], a reversible adhesion is desirable, and animals such as geckos are observed to use direction to switch from strong to weak adhesion [55]. This direction-dependent adhesion is attributed to the angled fibrils on the gecko’s foot, as evidenced by 20° cryo-SEM imaging [56]. While several methods were explored to fabricate vertical structures (e.g., e-beam lithography [57], nano-molding [58], constructing polymers from stiff thermoplastic [59], nano-drawing of stretched polymers [60], and growth of carbon nanotubes [61,62]), it is a challenge to obtain angled structures with high resolution and high aspect ratio, though several attempts were made (e.g., directional exposure in the lithography [63,64], deforming the shape memory polymer of vertical structures from soft lithography [65], post directional e-beam exposure of Pt-coated vertical polyurethane acrylate (PUA) nanohairs [66], and direct laser writing [67]). In another effort to address this challenge, an angled etching technique was developed, where a Faraday cage introduced in the conventional plasma etching system allows vertical movement of ions to induce an angled etching to the silicon substrate that is placed on an inclined stage [9]. Curing the polymer (e.g., polyurethane acrylate resin) in the etched Si master yields slanted structures with the designed angle and aspect ratio (Figure 1D). Taken together with the ultraviolet (UV)-assisted capillary force lithography, the etched Si master can be further used to create two-level hierarchical PUA hairs for enhanced robustness to a rough surface (<20 μm). In addition to the hierarchical structure [9,68,69], the shape of the tip was also found to have an influential role on adhesion strength [70] (e.g., mushroom-like and spatula tips [50,64,71,72] were shown to have higher adhesion than flat and round tips [73]).

**Figure 2:**
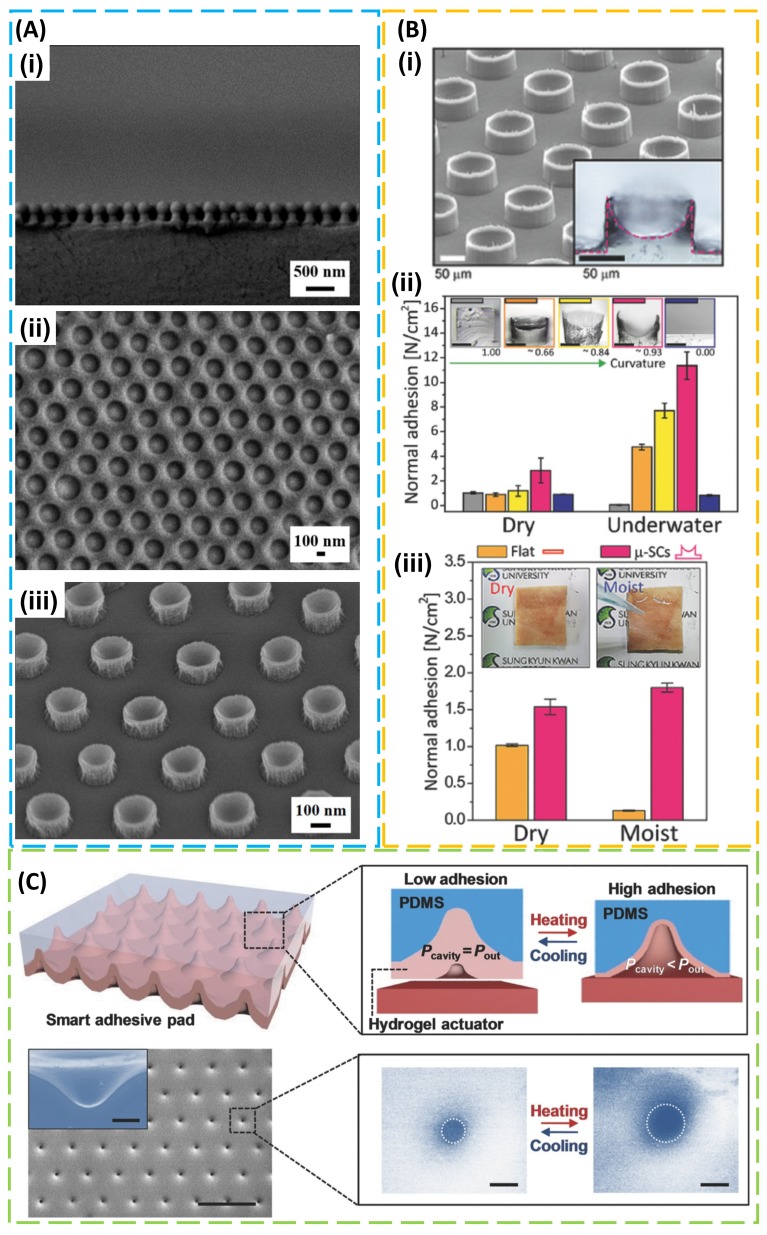
Octopus-inspired adhesive. (**A**) SEM images of (**i**) mushroom-like structures consisting of silica caps and polymer stems on a silicon wafer prepared by plasma etching; (**ii**) a polyvinyl alcohol (PVA) composite film embedding silica nanoparticles; (**iii**) a polydimethylsiloxane (PDMS) nano-sucker array obtained from the PVA composite mold. Reproduced with permission from Reference [84]; Copyright 2017, American Chemical Society. (**B**) (**i**) SEM image and cross-sectional optical image of micro-suckers (100 µm in diameter and 75 µm in height); (**ii**) dry/wet adhesion strength increases as the curvature of the cross-sectional profile increases; (**iii**) pull-off strength for PDMS-based micro-suckers and a non-patterned PDMS patch against a rough pigskin, with dry and moist pigskins shown in the inset. Reproduced with permission from Reference [85]; Copyright 2018, John Wiley and Sons. (**C**) Schematic representation and SEM image (scale bar: 10 μm) of micro-cavity arrays within a smart adhesive pad and its corresponding switchable adhesion mechanism (i.e., temperature-dependent hydrogel actuation in response to the environmental temperature). The inset in the bottom right shows SEM images of a single hole on the smart adhesive pad at a low temperature (left, T ≈ 3 °C) and a high temperature (right, T ≈ 40 °C) (scale bar: 500 nm). Reproduced with permission from Reference [85]; Copyright 2018, John Wiley and Sons.

On a separate route to structural design for enhanced adhesion, octopus suckers that reversibly adhere to wettable surfaces provided another source of inspiration [74,75,76,77]. The strong adhesion in both dry and wet environments results from the lower pressure in the octopus suckers than that of the environment. Using an external control (e.g., suctioning system [78], vacuum pump [79], dielectric elastomer actuator [80], or magnetic actuated film [81]), the biomimetic system can be easily created. The miniaturization of the system was also achieved through the use of lithographic processes [82,83]. In one attempt to create nanoscale suction cups [84], a non-close-packed self-assembled silica nanoparticle array served as an etching mask to prepare mushroom-like structures consisting of polymer stems and silica caps (Figure 2A (i)). Drying and peeling a polyvinyl alcohol (PVA) film from the etched structure created a replica with embedded silica nanoparticles (Figure 2A (ii)), which formed a mold to yield silicone polymer with nano-sucker structures (Figure 2A (iii)). By controlling the meniscus of a liquid precursor from applied pressure, a simple molding process from the mold with different surface energies could yield artificial micro-suckers with well-controlled cross-sectional profiles [85]. The adhesion in both dry and underwater environments was shown to increase as the curvature of the cross-sectional profile increased, due to the increased contact area from the preload (Figure 2B). In the wet environment, a model that combined the suction effect and capillary interaction [86] captured the experimental observation. When the elastomeric PDMS film with suction-cup structures was covered by thermoresponsive hydrogel of poly(*N*-isopropylacrylamide) (pNIPAM), a resulting smart adhesive pad could respond to temperature change, with an increased temperature inducing an increased volume and decreased pressure in the suction cup due to the deformation of the pNIPAM layer (Figure 2C) [87].

## 3. Design of the Material for Use in Wet Conditions

**Figure 3:**
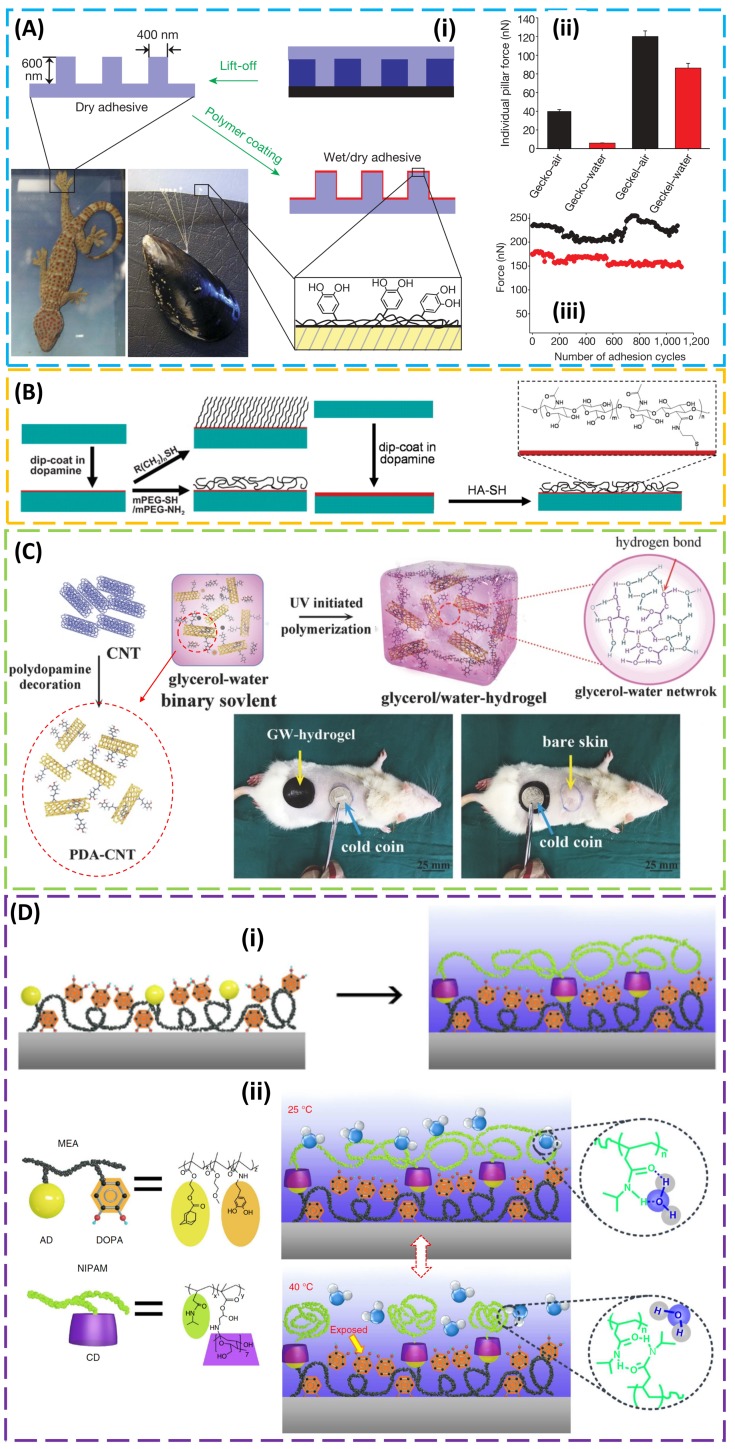
Mussel-inspired wet adhesives. (**A**) (**i**) PDMS casting onto the master is followed by curing, and lift-off results in gecko-foot-mimetic nano-pillar arrays. Next, the fabricated nanopillars are coated with a mussel-adhesive-protein-mimetic polymer that contains catechols, a key component of wet adhesive proteins found in mussel holdfasts. (**ii**) Adhesion force per pillar for gecko and geckel in water (red) and air (black). (**iii**) Performance of geckel adhesive during multiple contact cycles in water (red) and air (black) with error bars to represent the standard deviation. Reproduced with permission from Reference [7]; Copyright 2007, Nature Publishing Group. (**B**) Schematic illustrations of alkanethiol monolayer and poly(ethylene glycol) (PEG) polymer grafting, as well as the glycosaminoglycan hyaluronic acid (HA) conjugation to polydopamine (PDA)-coated surfaces. Reproduced with permission from Reference [7]; Copyright 2007, American Association for the Advancement of Science. (**C**) Polydopamine decorating carbon nanotubes (PDA-CNTs) in water results in PDA-CNT dispersion. Copolymerizing acrylamide (AM) and acrylic acid (AA) monomers in the PDA-CNT dispersion forms a glycerol–water (GW) hydrogel in a glycerol–water binary solvent. Frostbite and burn models on rats’ back skin demonstrate anti-freezing and anti-burning properties of the adhesive GW-hydrogel. Reproduced with permission from Reference [102]; Copyright 2018, John Wiley and Sons. (**D**) (**i**) Schematic diagram showing the specific procedure to prepare the wet adhesive, where dip-coating an adhesive guest copolymer poly(3,4-dihydroxy-l-phenylalanine) (pDOPA)/adamantine (AD)/methoxyethyl acrylate (MEA) on a clean Si substrate is followed by the self-assembly of host copolymer poly(*N*-isopropylacrylamide) (pNIPAM)/cyclodextrin (CD) using host–guest molecular recognition. (**ii**) Schematic drawing showing the tunable wet adhesion that responds to a local temperature trigger. When the local temperature of the adhesive is below lower critical solution temperature (LCST), the pNIPAM easily forms intermolecular hydrogen bonding with adjacent water molecules, and the infused water layer transforms the pNIPAM side chains to a swelling layer, which spatially stabilizes and confines the underlying adhesive moiety, DOPA. On the other hand, heating above the LCST leads to a phase transition and the collapse of pNIPAM-CD chains to form numerous agglomerates, exposing the adhesive group. Reproduced with permission from Reference [10]; Copyright 2017, Nature Publishing Group.

Though mechanical properties (e.g., the previously discussed structural designs and Young’s modulus of the structure [88,89,90]) showed significant effects on the strength of adhesion in the dry environment, many of them are compromised in the wet environment. In order to address the challenge, several bio-inspired materials [91,92,93] and their integration with structural designs [7,94] were explored. As a celebrated biological model for wet adhesion [95], mussels were shown to attach virtually all types of inorganic and organic surfaces, including classically adhesion-resistant materials such as poly(tetrafluoroethylene) (PTFE). Clues to this versatility may lie in the amino-acid composition of the specialized adhesive proteins that contain the catecholic amino acid 3,4-dihydroxy-l-phenylalanine (DOPA) and lysine [96]. DOPA and other catechol components perform well as adhesives. With inspiration from both geckos and mussels, a flexible organic nano-adhesive “geckel” was created by dip-coating the gecko-foot-mimetic PDMS pillar array in an ethanol solution of mussel-adhesive-protein-mimetic polymer (Figure 3A (i)) [7]. With a high catechol content, the adhesive monomer, dopamine methacrylamide (DMA), was used in a free-radical polymerization to synthesize poly(dopamine methacrylamide-co-methoxyethyl acrylate) (p(DMA-co-MEA)) as the mussel-adhesive-protein-mimetic polymer. The addition of a p(DMA-co-MEA) coating on the pillars enhanced the wet adhesion by nearly 15 times (Figure 3A (ii)), and this geckel nanoadhesive maintained its adhesive performance for over 1000 contact cycles in both dry and wet environments (Figure 3A (iii)).

Containing both catechol (DOPA) and amine (lysine) functional groups, dopamine as a simple-molecule compound also shows promise to achieve adhesion to a wide spectrum of materials [97]. The adherent polydopamine (PDA) coating produced by self-polymerization of dopamine can also serve as a versatile platform to graft various organic molecules and biomacromolecules for secondary surface-mediated reactions (Figure 3B). Taken together with its biocompatibility and hydrophilicity, polydopamine-based materials demonstrated great potential toward biomedical applications, ranging from cell adhesion/encapsulating/patterning to tissue engineering and re-endothelialization of vascular devices [98,99]. As a versatile building block, PDA was also integrated with other materials such as a hydrogel. However, hydrogel is often associated with long-term instability from water evaporation and physical changes from use at relatively extreme temperatures [100,101]. In order to provide hydrogel with long-term stable operation in a wide temperature window, a glycerol–water (GW) mixture as the binary solvent was used in hydrogel development (Figure 3C) [102], as glycerol is a well-known nontoxic anti-freezing agent. Incorporating PDA-decorated carbon nanotubes (CNTs) as conductive nano-fillers into hydrogel imparts good conductivity (~8 S/m), enhanced toughness (~2000 J/m^2^), and excellent adhesion (57 kPa to porcine skin) to the resulting GW hydrogel. Due to its advantages of good adhesiveness and anti-heating or anti-freezing properties, GW hydrogel demonstrated its capability to protect skin from damage in harsh environments (e.g., during frostbite or burn) by serving as an excellent wearable dressing. Other challenges of hydrogel also include foreign body response and poor mechanical properties (i.e., toughness and stretchability). The former could be attenuated by encapsulating mesenchymal stem cells within the hydrogel, such as poly(ethylene glycol) (PEC) [103], and the latter is addressed by the design of tough hydrogel [104,105,106], discussed in Section 4.

In order to provide reversible and tunable wet adhesion that responds to a local temperature trigger in an on-demand manner, a mussel-inspired guest-adhesive copolymer was combined with a thermoresponsive host copolymer [10]. The guest copolymer pDOPA/adamantine (AD)/methoxyethyl acrylate (MEA) consists of a mussel-inspired adhesive DOPA polymer, a guest motif adamantine (AD), and a methoxyethyl acrylate (MEA) monomer as a hydrophobic matrix to enhance the wet adhesion of DOPA (Figure 3D (i)). In the host copolymer pNIPAM/cyclodextrin (CD), the poly(*N*-isopropylacrylamide) (pNIPAM) undergoes a reversible lower critical solution temperature (LCST) phase transition from a swollen hydrated state to a shrunken dehydrated state when heated above the LCST and β-cyclodextrin (β-CD) is the host molecule providing selective binding with the AD moiety in the guest copolymer. Dip-coating the as-prepared guest copolymer on the target substrate surface (e.g., Si, Ti, Al, glass, PTFE, or PDMS) allows the self-assembly of the host copolymer through the host–guest interaction. When the local temperature of the adhesive is below the LCST, the swollen hydrated pNIPAM spatially confines and stabilizes the underneath adhesive moiety, DOPA, through the host–guest interaction, resulting in a dramatically screened interaction area and reduced adhesion. In contrast, the collapsed pNIPAM exposes the adhesive moiety, DOPA, when the local temperature is above LCST (Figure 3D (ii)). The versatile demonstration of the wet adhesive also goes from inorganic (Si, Ti, Al, and glass) to organic surfaces (PDMS and PTFE). In addition, the gecko-like surface structure (e.g., an array of PDMS posts with a diameter of 5 μm and and height of 10 μm), discussed in Section 2, was explored to further enhance the interfacial adhesion strength, which is in direct contrast with the gecko-like dry adhesive.

Although mussel-like wet adhesion was successfully realized, typical catechol functionalization and solution processing entail complex components and steps. In order to reduce the complexity, synthetic low-molecular-weight catecholic zwitterionic surfactants were developed to adhere to diverse surfaces with very strong adhesion (~50 mJ/m^2^) [107]. Based on catechol-modified amphiphilic poly(propylene oxide)/poly(ethylene oxide) (PPO-PEO) block copolymers, a mechanically tough zero- or negative-swelling mussel-inspired surgical adhesive was synthesized, minimizing the weakening mechanism from swelling [108]. The range of zero to −25 % swelling was achieved through a hydrophobic collapse of PPO blocks upon heating to physiological temperature. The lap shear adhesion measurements of decellularized porcine dermis show nearly 50 kPa adhesive strength. Although the single-layer mussel-like adhesion is effective, a layer-by-layer (LbL) assembly may be explored to further enhance the adhesion strength due to the versatile control in the assembly process (e.g., introducing sodium chloride in the assembly process yields an adhesion enhanced by two orders of magnitude [109]).

## 4. Adhesion to Biological Tissues

**Figure 4:**
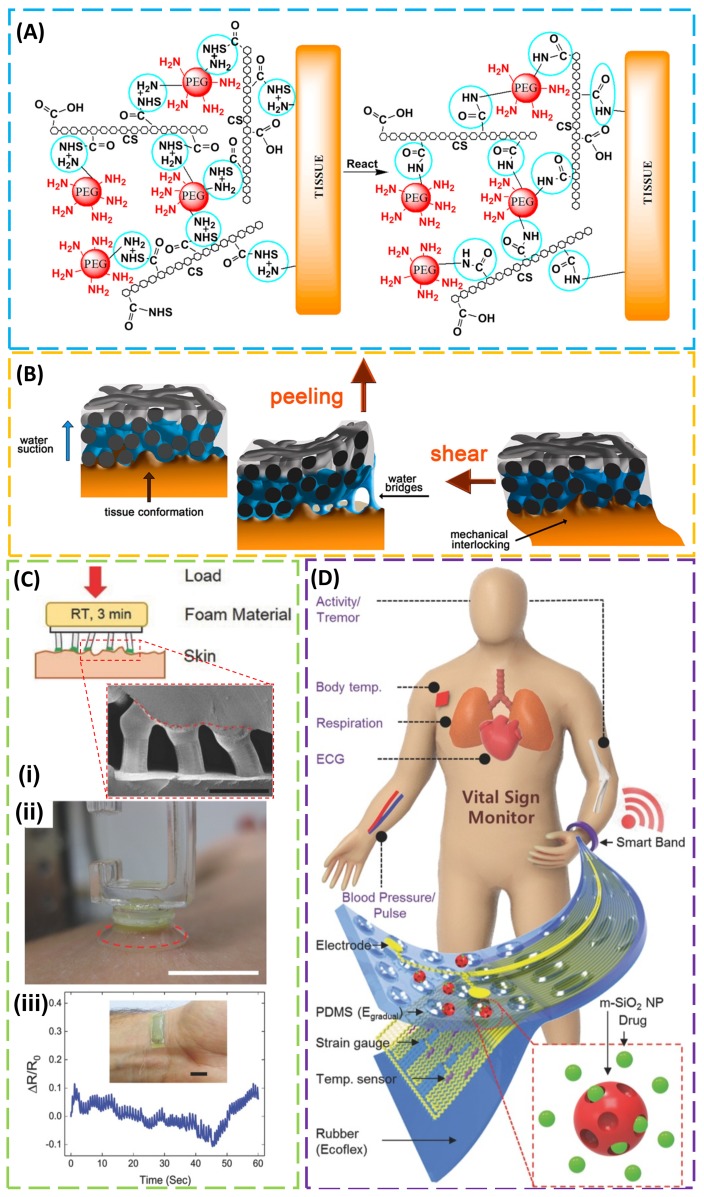
Adhesives on soft tissues. (**A**) Chondroitin sulfate *N*-succinimidyl succinate (CS–NHS) reacts with primary amines of both six-arm polyethylene glycol amine PEG–(NH_2_)_6_ and proteins of the tissue to form a covalently bonded hydrogel to the tissue. Reproduced with permission from Reference [115]; Copyright 2010, Elsevier. (**B**) When the buckypaper (BP) comes into contact with the wet biological tissue, water suction leads to deformation of the soft tissue and conformal contact of the BP to the tissue. The resulting water bridge formation and BP–tissue mechanical interlocking help enhance the adhesion during peeling and shearing tests. Reproduced with permission from Reference [116]; Copyright 2013, American Chemical Society. (**C**) (**i**) Inking vinylsiloxane (VS) precursor onto uniformly shaped cylindrical microfibers followed by curing vs. directly onto the skin surface. The inset shows the cross-sectional SEM image of an adhesive film attached to an artificial skin replica, where the red dashed line indicates the interface between the skin-adhesive film and artificial skin. (**ii**) Optical image of a skin-adhesive film attached to the human forearm during the retraction cycle of the adhesion experiment (dashed red line indicates the interfacing border between the adhesive film and the skin; scale bar: 1 cm). (**iii**) The output signal from a microfibrillar strain sensor mounted onto the radial artery of the wrist; the inset shows a photograph of the strain sensor attached to the wrist through the inking and printing process. Reproduced with permission from Reference [26]; Copyright 2017, John Wiley and Sons. (**D**) Schematic illustration of the multifunctional electronic patch integrated onto an octopus-inspired adhesive layer with miniaturized suction cups (mSCs). The multifunctional electronic patch is capable of monitoring vital signs from electrodes, strain gauges, and temperature sensors, as well as transdermally delivering drugs loaded in mesoporous silica nanoparticles via iontophoresis. The smart band (connected to the mSC patch) is used to provide wireless functionalities and a power source. Reproduced with permission from Reference [83]; Copyright 2015, John Wiley and Sons.

When it comes to adhesion to biological tissues such as skin, several additional challenges are encountered, including soft properties, multiscale roughness, and biocompatibility. For instance, adhesives based on chemical bonding may irritate the skin and cause discomfort upon removal due to strong adhesion. Though several commercial adhesives were used in bio-integrated electronics on the skin [110,111], their applications are limited by their given properties, and the adhesion strength was also shown to be dependent on the target tissue. Taking a synthetic tissue adhesive (i.e., Dermabond^®^, 2-octyl cyanoacrylate) as an example, its adhesion to collagen films was observed to be 40 times that when compared with its adhesion to muscle tissue, due to increased wetting (and the decreased contact angle) of the Dermabond^®^ adhesive on the collagen film [12]. In the two classes of tissue adhesives, biologic (e.g., fibrin glue) and synthetic (e.g., *n*-butyl-2-cyanoacrylate) [112], a variety of different bonding mechanisms were explored (e.g., physical interaction, mechanical interlocking, and chemical bonding) [113,114]. As an extensively used synthetic polymer for tissue engineering, polyethylene glycol (PEG) was used with chondroitin sulfate (CS) to form a biodegradable CS–PEG adhesive hydrogel that can covalently bond to proteins in tissue or to collagen in the extracellular matrix via amide bonds, improving the adhesion strength by ten times that of fibrin glue (Figure 4A) [115]. In a separate effort, a buckypaper (BP) film produced from oxidized multi-walled carbon nanotubes demonstrated enhanced adhesion to the rimmed muscular fascia of the abdominal wall of New Zealand female rabbit during both peeling and shearing tests, due to soft tissue deformation from water suction resulting in water bridge formation and BP–tissue mechanical interlocking, respectively (Figure 4B) [116].

Because of the self-cleaning effect and adaptation capability, the gecko-inspired fibrillar adhesive demonstrated repeatable and restorable adhesion to the skin surface over multiple cycles of use [5]. Though the adhesion does not show a direct correlation with classical roughness parameters, strong adhesion was shown to decrease significantly when surface roughness increased [117,118,119] and a clear correlation was even observed when a newly integrated roughness parameter was introduced [120]. In order to address these challenges, special considerations have to be given to the design of the adhesion layer. In one effort to utilize the unique advantages of the gecko-inspired fibrillar adhesive, a composite adhesive was designed by coating polymer microfibers with a skin interfacing material (e.g., vinylsiloxane) to form mushroom-shaped tips (Figure 4C) [26]. As a member of the family of silicone rubbers, the biocompatible vinylsiloxane (VS) approved for biomedical applications (e.g., forming dental impression) can fully cross-link and form covalent bonds with the PDMS microfibers within a few minutes at room temperature, enabling its direct cross-linking on and conformal contact to the skin surface with multiscale roughness. Due to the high flexibility and strong attachment to the skin surface, the adhesive layer shows an efficient strain signal to the strain sensor integrated on top, thereby significantly increasing the signal-to-noise ratio when compared with medical tape or fibrillar adhesive film fully immersed into a flat vs. film. The adhesion strength of the composite adhesive to a wet skin surface was also shown to be comparable to that of a dry environment. In contrast to the gecko-inspired adhesive, the octopus-inspired adhesive that relies on the pressure difference was less affected by the surface roughness. Such an adhesive also showed comparable adhesion strength to pigskin in moist conditions (40% of area covered with droplets) to that in dry conditions, even when hairs were present [85]. When integrated with physiological sensors and drug-delivery actuators, the octopus-inspired adhesive allowed sensitive biometric measurements and transdermal drug delivery through tight skin coupling (Figure 4D) [83].

**Figure 5:**
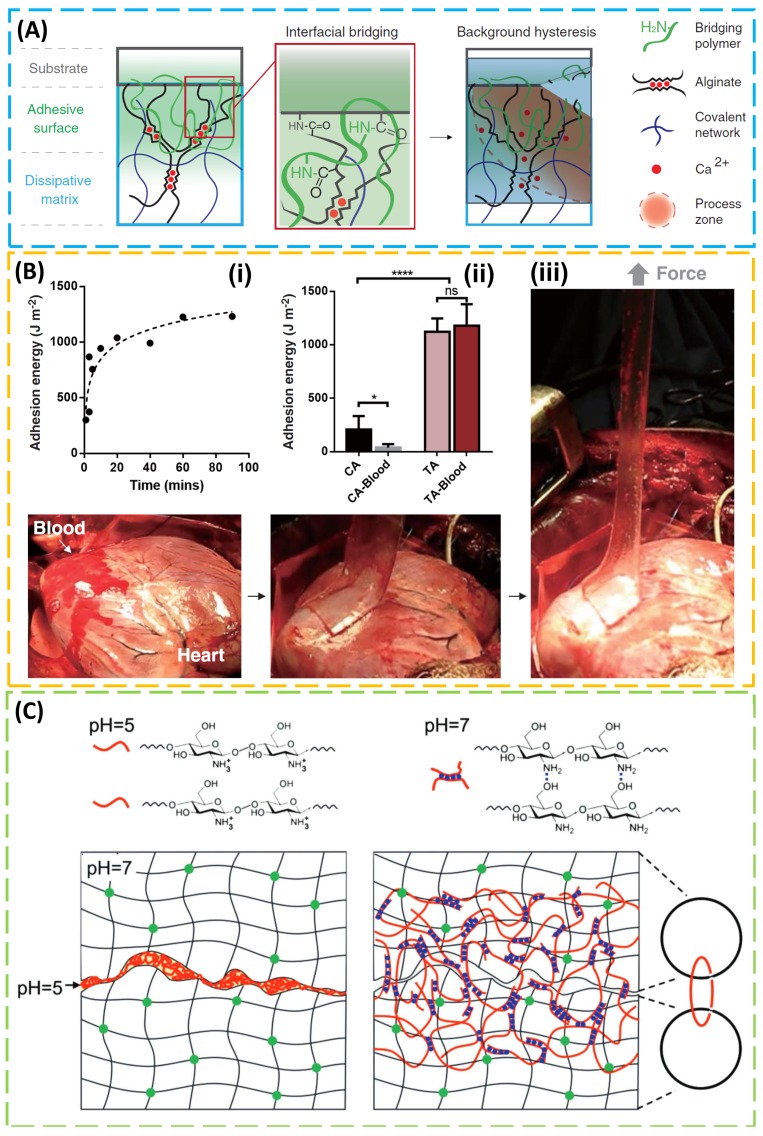
Tough and topological adhesives. (**A**) Tough adhesives (TAs) consist of a dissipative matrix (light-blue square), made of a hydrogel containing both ionically (calcium; red circles) cross-linked and covalently cross-linked polymers (black and blue lines), and an adhesive surface that contains a bridging polymer with primary amines (green lines). The bridging polymer can penetrate into the TA and the substrate (light-green region) to facilitate covalent-bond formation. In the presence of a crack, the process zone (orange area) dissipates significant amounts of energy as ionic bonds between alginate chains and calcium ions break. (**B**) (**i**) Tough adhesives exhibit a rapid increase in adhesion energy to porcine skin over time. (**ii**) Comparing with cyanoacrylate (CA), TAs showed strong adhesion even when the porcine skin was exposed to blood in the in vitro experiment. *n* = 4–6. (**iii**) The TAs were further tested in an in vivo experiment on a beating porcine heart with blood exposure. (**A**,**B**) Reproduced with permission from References [105]; Copyright 2017, American Association for the Advancement of Science. (**C**) Chitosan chains dissolve in water at pH 5 and form a network in water at pH 7. Placing an aqueous solution of chitosan of pH 5 between two hydrogels (or biological tissues) of pH 7 is followed by the diffusion of chitosan chains into the two hydrogels, forming a network that topologically entangles with the networks of both hydrogels. Reproduced with permission from Reference [125]; Copyright 2018, John Wiley and Sons.

Relying on relatively weak physical interactions, existing tissue adhesives (including mussel-inspired adhesives) are associated with low adhesive energy on the order of 10 J/m^2^ [121], which is far from ideal, especially when compared with the example in nature. For instance, cartilage bonds to bones with an adhesion energy of 800 J/m^2^ [122]. In order to achieve high adhesion, a synergy from an adhesive surface layer and a dissipative matrix was explored (Figure 5A) [105]. The design was inspired by a sticky and tough secretion from slug *Arion subfuscus* [123], which may arise from two interpenetrating networks of polymers [124]. The strong adhesion from the adhesive surface layer to the tissue substrate can be achieved through electrostatic interactions, covalent bonds, or physical interpenetration. Meanwhile, the energy dissipation through hysteresis in the matrix amplifies the effective adhesion energy. As the surface of tissues or cells is negatively charged, a bridging polymer that bears positively charged primary amine groups enables covalent binding via electrostatic attraction. In the case of a permeable target surface, the bridging polymer penetrating into the target forms a physical entanglement and a chemical anchor for the adhesive. As for the dissipative matrix, a substrate that can dissipate energy is used. By exploiting the synergy of these two factors, a class of tough adhesives demonstrated high adhesion energy (~1000 J/m^2^) on wet surfaces. In vivo demonstrations included strong adhesion to a beating porcine heart in the presence of blood, heart sealants to prevent liquid leakage, and hemostatic dressing for a deep wound (Figure 5B). This simple yet effective strategy opens up a wide range of applications, including tissue adhesives, wound dressing, and tissue repair.

In order to provide a reversible adhesion that can respond to the external stimuli, a responsive polymer was explored to diffuse into and form an entangled network with two polymer networks of two wet materials for enhanced adhesion upon the trigger of one signal (e.g., in one pH range), while being soluble and separating both wet materials upon trigger removal (e.g., in the other pH range) (Figure 5C) [125]. In the demonstration, several stitching polymers were identified to cover the full range of pH (e.g., cellulose forms a network for pKa < 13, alginate for pKa < 3.5, chitosan for pKa > 6.5, and poly(4-aminostyrene) for pKa > 4.5). Adhesion energy as high as 1000 J/m^2^ could be achieved when the stitching polymer introduced hysteresis in the wet hydrogel materials. The demonstrated strong adhesion also went beyond the hydrogel to various porcine tissues (e.g., skin, liver, heart, artery, and stomach) and the skin was shown to exhibit a relatively high adhesion energy (100 J/m^2^) due to its relatively high toughness. 

## 5. Conclusions and Future Perspectives

In order to robustly adhere bio-integrated devices to soft biological tissues, an adhesive layer with tunable adhesion is of great interest. The capability to switch between strong and weak adhesion would allow the use of strong adhesion to efficiently transfer vital signals to the device for accurate measurement, followed by the use of weak adhesion for easy removal. However, this tunable adhesion to the tissue surface has long been a challenge due to multiscale roughness, wet conditions, biocompatibility, and natural motion, among many others. Thanks to the recent developments that shed light on the underlying mechanisms of the remarkable adhesion observed in several animal species, great strides were made, and effective strategies ranging from structural design for dry adhesion to novel material synthesis for wet conditions were explored to yield adhesives that can match or even outperform those from nature. The importance of the developed adhesives also goes beyond bio-integrated devices to cell culture [126,127] and to tissue glues that can potentially replace sutures in clinical practice. When combined with the tunable properties of the adhesive, tissue glues would promise repeatable use, which can dramatically reduce the cost and pave the way for commercialization. Despite great strides made in the field of tissue adhesives, several challenges still exist, including fabricating high-aspect-ratio fibrillar structures with diameters down to submicron scales [40], long-term reliability of the tissue adhesives to diverse wet surfaces, tunable properties in the adhesives to accommodate dynamic changes in target tissues, and integration with multifunctional electronics for real-time sensing and closed-loop control [128,129]. In the burgeoning field of tissue adhesives, different testing methods and tissue models are used to evaluate the adhesive properties of newly developed structures and materials. Thus, it is a bit challenging to directly compare the results reported by different research groups. It would be desirable to have standardized testing procedures and tissue models in place to allow for direct comparison among the newly developed tissue adhesives. Nevertheless, the challenges simply represent a small fraction of the great opportunities for future development, which may require the collective wisdom of material scientists, chemists, mechanical engineers, and clinicians, among many others.
